# 2023 Industry Perceptions Survey on AI Adoption and Return on Investment

**DOI:** 10.1007/s10278-024-01147-1

**Published:** 2024-08-20

**Authors:** Mitchell Goldburgh, Michael LaChance, Julia Komissarchik, Julia Patriarche, Joe Chapa, Oliver Chen, Priya Deshpande, Matthew Geeslin, Nina Kottler, Jennifer Sommer, Marcus Ayers, Vedrana Vujic

**Affiliations:** 1 NTT DATA, Tokyo, Japan; 2 HOPPR, Chicago, IL USA; 3 Department of Electrical and Computer Engineering, Opus College of Engineering, Marquette University, Milwaukee, WI USA; 4 Deparment of Radiology, University of Vermont, Burlington, VT USA; 5 Glendor, Inc., Draper, UT USA; 6 Radiology Partners, El Segundo, CA USA; 7 A.I. Analysis, Inc., Seattle, WA USA; 8 Department of Radiology, University Hospitals Cleveland Medical Center, Cleveland, OH USA; 9 LaChance Executive Consulting, Washington, DC USA

**Keywords:** Artificial Intelligence, AI Adoption, Market Survey

## Abstract

This SIIM-sponsored 2023 report highlights an industry view on artificial intelligence adoption barriers and success related to diagnostic imaging, life sciences, and contrasts. In general, our 2023 survey indicates that there has been progress in adopting AI across multiple uses, and there continues to be an optimistic forecast for the impact on workflow and clinical outcomes. This report, as in prior years, should be seen as a snapshot of the use of AI in imaging. Compared to our 2021 survey, the 2023 respondents expressed wider AI adoption but felt this was behind the potential. Specifically, the adoption has increased as sources of return on investment with AI in radiology are better understood as documented by vendor/client use case studies. Generally, the discussions of AI solutions centered on workflow triage, visualization, detection, and characterization. Generative AI was also mentioned for improving productivity in reporting. As payor reimbursement remains elusive, the ROI discussions expanded to look at other factors, including increased hospital procedures and admissions, enhanced radiologist productivity for practices, and improved patient outcomes for integrated health networks. When looking at the longer-term horizon for AI adoption, respondents frequently mentioned that the opportunity for AI to achieve greater adoption with more complex AI and a more manageable/visible ROI is outside the USA. Respondents focused on the barriers to trust in AI and the FDA processes.

## Introduction

The definition of artificial intelligence and the expectations that it will be widely adopted in applications for radiology and medical imaging, in general, continue to drive interest from investors, engage professionals around guidelines for adoption, and have a dominant content in contribution.

The Society for Imaging and Informatics in Medicine (“SIIM”) engaged with the industry to explore opinions on AI adoption, the return on investment from artificial intelligence, and the future adoption of artificial intelligence in medical imaging. This report summarizes the process and the results.

## Methodology

For the second year studying opinions on artificial intelligence (“AI”) from industry, SIIM sent invitations to companies in life sciences, imaging modality vendors, Picture Archive and Communication System (“PACS”) vendors, and AI vendors in the USA and abroad.^1^ The targets for participation were without any limitations that participants be corporate or individual members of SIIM.

The 30-min confidential discussions for this initiative were conducted in early 2023 by members of a dedicated SIIM committee of representatives across industry and providers. The discussion guide has consistently had three categories of discussion, and the detailed report analysis follows the following order of topics:

1. Context: respondents and their position, and the company’s role in AI for imaging
2. Current state of AI: respondents were asked in open-ended questions about the state of imaging AI today, including barriers to adoption.
3. Future of AI: respondents were asked to share their how they envision imaging AI evolving in the next 2–5 years for their products and for the healthcare/life science industry overall.

As a discussion guide, the three categories each had example questions developed by the research team but interviewers were free pursue topics based on the responses.

Best efforts were made to have each interview session have two interviewers, one with prior experience in this process and a recorder who took notes of the discussion. Committee members did some specific categorical encoding of the unstructured responses based on notes and some background research on the participant’s companies. In 2023, the data represents content from 36 interviews (a 30% increase from the prior survey in 2021).

## Respondent Overview

This survey outreached to contacts who were directly involved in the development or deployment of AI at various companies, as illustrated in the tables below. The response rate for the solicited companies was 100% once the company contact was made. As the breadth of companies by size and maturity differed, we have consolidated the titles for a more thoughtful view.

In comparison, 2021’s respondent population had a slightly stronger voice from the sales role representation in the respondent pool. Comparable to the prior collection of respondents, there was a mix of interviews with product leads and overall product management. Also noted were the respondents’ roles, including sales persona in 2021. In contrast, in 2023, the voices were more strategic or tactical in their commentary. The authors considered that this may have influenced our summation of market maturation and the broader audience in 2023.

Table 1 summarizes the companies represented in the interviews. The content for these summaries was generally defined based on a review of commercially available materials on organizational capabilities/offerings. The duplicates on organizational ability recognize that although we spoke with individuals within organizations, the overall capabilities may have extended beyond the respondents’ department. For example, PACS companies now offer AI markets and deployment engines, and AI companies offer both AI and platforms for their specific AI. The clinical category references the respondent’s organization as a hospital.

**Table 1:** 2023 respondent organization category

**Organization category (company can belong to several categories)**	**Count**	**Corporate nationality**	**Count**
Healthcare IT^a^	13	North America	15
**AI** platform	13	Multinational	13
**AI** vendor	12	Europe (incl. UK)	5
Big iron^b^	7	Asia-Pacific	2
**PACS/RIS/VNA**	4	Middle East	1
**Clinical**	2		
**Pharma**	2		

^a^This is, in a sense, a catchall for companies that are not any of the other categories and includes the likes of dictation, image exchange, etc. So, companies that had a portfolio of offerings beyond AI and PACS were included here as well

^b^Big Iron refers to those companies that offer a broad range of core imaging modalities in x-ray, magnetic resonance, computed tomography, and other domains. If a Big Iron vendor also offers a PACS then it is assigned both Big Iron and PACS category

The authors researched and made assignments of categories in Tables 2 and 3, providing for cross-correlation of the respondents and their organizational categories. The intent was to highlight possible influences in the perspectives shared in the subsequent interview findings. The report team also assigned company sizes (large over $100 M, medium over $50 M, and small under $50 M) to each organization based on public information on total healthcare revenue. This categorization led to the conclusion that 14 of the 36 participating companies would be considered large organizations. This company size and market footprint add different views to the standalone AI companies that participated with less extensive staff and revenue. Future surveys will continue to explore any bias in market views based on organizational mix and size characteristics.

**Table 2:** 2023 interviewees’ job titles categorized by organization category

	**Organization category**						
**Job title**	Healthcare IT	AI platform	AI vendor	Big Iron	PACS/RIS/VNA	Clinical	Pharma
**CXO**^a^	8	11	9	2	2	2	1
**VP/head**	4	2	2	2	2		1
**Manager**	-	-		2	-	-	-
**Director**	1	-	1	1	-	-	-
**Total**	13	13	12	7	4	2	2

^a^Titles include chief marketing officer, chief revenue officer, chief executive officer, and chief technology officer

**Table 3:** 2023 interviewees’ background and focus (multiple assignments)

**Respondents background**^a^	**Count**	**Respondent job focus**	**Count**
**Clinical medicine**	13	Strategy	22
**Engineering**	10	AI development	15
**Science**	7	Management	13
**Strategy**	6	Product management	6
**Academia**	5	Science	5
**Marketing**	4	Clinical practice	4
**Product management**	3	Engineering	3
**Sales**	1		
**Government**	1		

^a^The source is the LinkedIn^®^ information “current at the time of the interview.” That is, some interviewers were promoted or changed companies by this time. So, in those cases the position at the time of the interview was used

## Types of AI Applications Offered by Participating Companies

The pool of respondents included vendors and users of different types of artificial intelligence related to medical imaging. The question prompting discussion was regarding the priority use cases our respondents are adopting or selling today and how they might see these priorities changing over the next 18 months. Table 4 summarizes the responses to this question, with multiple responses allowed per interview.

**Table 4:** 2023 priority use cases: today and looking out 18 months

**Priority use cases: today and looking out 18 months (*multiple choice allowed*)**	
**Workflow automation**^a^	23
**Diagnostic assistance**	19
**Triage**	15
**Analytics**	5
**Report generation**	4

^a^Often imaging operations, but also multimodality workflow automation

Overall, the respondents expressed that AI specific to imaging interpretation remains a maturing market opportunity. The ability or inability to describe a ROI remains top of mind. Notably, the new 2023 respondent pool included radiologists and vendors involved in the reporting with AI solutions for radiology studies to pre-populate radiologist reports, as noted in Table 4.

Consistently across both surveys, four categories of AI were tracked across surveys—workflow triage, visualization, detection, and deterministic characterization. This reflects that many of the companies from the 2021 survey also participated in the 2023 and not necessarily a predominance of applications in the market.

Mention of the future perceived trends was aligned around the growing capabilities of AI. Table 5 highlights two categories of mention forecast over the next 18 months and beyond. Examples of imaging AI discussed in the interviews included AI to automate diagnostic annotation (e.g., mammography, neurology, oncology) where adoption is high. In comparing interviews, mentions of AI growing in breadth and depth include vendors harnessing more AI input to assist with report generation and modality vendors expanding the use of AI in image reconstruction.

**Table 5:** Mentions of perceived trends in 2023 interviews

**Perceived trends**	
**Multiple algorithms in one package**	12
**Adoption of multi-modality**	9

In 2023, some respondents discussed the advantages of the different delivery mechanisms for adopting AI as part of PACS, standalone AI platforms, or combinations of solutions. Selected respondents who addressed the emerging delivery models suggested that, in some cases, the platform reduced barriers versus implementing individual AI solutions. The 2023 survey included more vendors working with platform companies that expressed this approach simplified technical deployment. This led to discussions about features and functions of adopting AI that were expanded from 2021 interviews. In 2021, AI was described as the “seatbelt” of PACS, and ROI relied only on reimbursement to insightful discussions about the market’s direction related to productivity and outcomes. In 2023, AI adoption discussions went deeper, and several respondents identified costs to implement and maintain AI as potential barriers to adoption and cost impact on ROI.

## Respondent View—Who Is the Customer for AI?

Between the two surveys (201 and 2023) from this committee, there was an evolution in the respondents’ view of their audience. While radiologists continue to be beneficiaries of AI, and thus, initially, all PACS came with some form of AI, in this recent survey, radiologists were not the buyers in hospital settings. As there are specific clinical champions within the hospital, there were several mentions of hospitals becoming engaged in the efficiencies brought about by AI adoption.

The 2023 survey respondents see AI adoption moving beyond academic facilities to more general hospitals and large teleradiology practices, as shown in Table 6 below. Respondents expressed that the audience for AI is large practices or integrated delivery networks with significant volumes in radiology as target customers. The absence of frequent mention of applications in life sciences and med device companies may be more about our vendor selection and pool than the overall market. Subsequent surveys may broaden participation to address this potential bias in responses.

**Table 6:** 2023 target customers: today and in 18 months

**Target customers: today and looking out 18 months (*multiple choice***)	
**Hospitals/labs/practices**	27
**Researchers**	4
**Pharma**	4
**AI in med device companies**	3
**Patient**	2
**Payers**	2

In describing the purchase process, multiple mentions of “steering committees” were made as part of the process gates toward contracting. In the latest survey, comments suggest such committees are composed of radiologists and IT, yet there is minimal reference to the involvement of referring providers or CMIOs. CIOs were also not the drivers of AI adoption.

Across the board, institutions are looking for investment vs. return, whether through productivity or reimbursement. Based on the breadth of respondents, we heard descriptions of ROI based on workflow for internal productivity within the department, changes to patient engagement, and patient follow-up that led to increased reimbursement for patient care, including increased volume from follow-ups.

Channels to market for independent AI companies included PACS companies, AI platforms, and AI marketplaces. Platforms describe their role in simplifying interoperability and integrating results. However, the implication is that this platform cost is now part of the ROI calculation.

## Respondent View—Current Sources of AI ROI

The discussions about ROI remained in the format of open-ended questions. This format is intended to allow respondents to describe the ROI from the perspectives of their company or product and/or the ROI of the customers. In this 2023 survey, many respondents were focused on the ROI from AI adoption for the customer, whereas in prior years, there were mentions such as “to sell PACS, we need to have an AI story.” This perhaps reflects either the more strategic roles of the 2023 respondents versus the sales roles in the prior survey or the growth in adoption with more mature ROI case studies being documented.

To set the context for the 2023 discussion on ROI, we show in Table 7 a summary of the mentions of sources for customer ROI. ROI from “efficiency,” perhaps inclusive of the term productivity, is nuanced by the mention of “reduced FTE.”

**Table 7:** 2023: source of ROI for AI for users of AI

**Source of ROI for AI for users of AI** ***(multiple choice)***	**Count**
**Efficiency improvement**	22
**Quality of care improvement**	10
**Reduce FTE**	6
**Improved sensitivity/specificity**	3
**Billing code**	3
**Reduced unnecessary tests, procedures**	2
**Reduced liability**	2
**AI will be required as standard practice**	2
**Added incidental findings**	1

Another slice of the data maps the terms into categories summarized in Table 8 that correlate the respondents’ roles with identifying ROI sources for their customers or consumers of AI for imaging. Table 8 is more about “how” the benefit in Table 7 is “packaged” or “messaged” by the respondent and our analysts for this report.

**Table 8:** 2023: source of ROI by job title

**Job title**	**Source of ROI for AI**				
	Workflow automation	Diagnostic assistance	Triage	Analytics	Report generation
**CXO**^a^	13	14	12	3	3
**VP/head**	6	3	2	2	1
**Manager**	2	1		-	-
**Director**	2	1	1	-	-
**Total**	23	19	15	5	4

^a^Titles include chief marketing officer, chief revenue officer, chief executive officer, and chief technology officer

Table 8 shows that the CXO responses generally were distributed when the company size allowed for breadth, whereas the AI companies were generally single “source of ROI” mentions. Similarly, even though titles of smaller companies were CEOs, across the board, the CXO respondents were involved in selling their platform and AI. Thus, the feedback we received is generally based on their direct interaction with the market and reflects the snapshot of where ROI is most perceived to be attained.

Table 9 below complements Table 1 of derived company profiles mapped to responses on ROI that again stress the focus on workflow automation followed by diagnostic assistance as the main sources of ROI—both being under productivity gains.

**Table 9:** Sources of ROI by organizational category

	**Source of ROI for AI**				
**Organization category**	Workflow automation	Diagnostic assistance	Triage	Analytics	Report generation
**Healthcare IT**	8	5	5	2	3
**AI platform**	5	9	7	2	2
**AI vendor**	7	6	5	2	2
**Big iron**	7	3	4	1	-
**PACS/RIS/VNA**	3	2	-	2	-
**Clinical**	1	1	2	-	1
**Pharma**	1	1	1	-	1

Relative to the mentions of reimbursement as an element of ROI, analogies related to reimbursement for advanced visualization analysis were brought up as a baseline reference, as were other CPT new technology codes.^2^ However, the discussion of establishing the justification and issuance of the CPT reimbursement process was deemed too long, and adoption by consumers of AI needed to be more specific, given the complex documentation required from the provider organization. However, there was mention that any long-term productivity gains may eventually be noticed by CMS and followed by subsequently reduced reimbursement, as was evidenced in the adoption of PACS.^3^

Table 10 specific terminology quoted from the responses is a raw summary of some phrases collected across the 36 interviews. In several cases, there was no clear category, and these were omitted.

**Table 10:** Specific terminology quoted from the responses

**Source of ROI**^a^ ***(multiple mentions allowed)***	**Count**
**Disease detection**	8
**Interoperability**	1
**Outcomes**	5
**Quality**	2
**Radiologist productivity**	10
**Scale of adoption**	3

^a^These quotable terms were categorized into summaries in Tables 9 and 10 above

The mention of non-workflow outcome-based models related to shorter hospital stays and projections of improved outcomes was mentioned as still a way off. Respondents projected that more concrete studies on the downstream impact of radiology AI would be coming as presentations in November, 2023, at the Radiology Society of North America annual event.

Productivity discussions included modality-specific and visualization tools. In this instance, the ROI was both technologist productivity or the number of patients scanned and benefits in lower dose, less contrast, and shorter scan times due to reconstruction AI.

The survey focused discussions on the context of image analysis (versus other types of AI for non-imaging purposes such as transcription), as this involves software as a medical device. Respondents commented thusly on the differences in vendor ROI versus ROI of consumers of AI.

In describing ROI for AI users, the path of savings from AI which had license costs per diagnostic finding was cited as productivity. Very few AI had fiscal compensation for the use of AI. Thus, the adoption and savings model was still most apparent in large healthcare organizations to cover the costs of licensing AI. In the USA this included teleradiology practices, US public health organizations, or public healthcare delivery models outside the USA. Respondents with AI product installed outside the USA had compelling stories of ROI from productivity based on volumes and wide adoption. Respondents felt adoption of AI had yet to scale in the US markets across the smaller vertical provider market customer segments (community and non-hospital organizations).

For vendors of AI, the ROI involves the costs of AI development and release for sale. In the USA, post-AI development, vendors incur costs for the registration of AI software as a medical device in the USA with FDA registration and/or international certification processes. Specifically with the FDA, for AI capable of multiple findings in a single study, may require multiple submissions to have multiple inferences. This was considered costly, imposing levels of efforts for AI developers, impacting the ability to achieve a short-term ROI and achieve scaling AI features for single studies where respondents felt consumers of AI tools would see the most benefit.

In comparing the responses to sources of ROI in 2023, the 2021 survey respondents used different terms to describe ROI, as shown in Table 11.

**Table 11:** Excerpts from the 2021 survey on AI ROI

**2021 mentions of ROI in 3–5 by role** (** denotes multiple mentions across roles)
**Role: Business development**
Productivity gains**
Long time to ROI**
Consolidation is coming**
Downstream benefits of using AI**
**AI is an experiment**
**Role: Product officer/manager**
**Productivity gains**
**Long time to ROI**
**Consolidation is coming**
**Automation of processes**
**Data monetization adoption**
**Role: President/sales**
** Long time to ROI**
**Consolidation is coming**
**Downstream benefits of AI**
**Imaging AI replacing intervention**
**Competitiveness leading to volume needing AI**
**Need reimbursement (CPT/billing)**
**Over investment by VC into AI leading to reduced investment going forward**
**Transformation of radiology from AI**
**Role: VP sales**
**Productivity gains**
**Wait and see on ROI as no direct reimbursement**

The terminology differences may have several causes; including in the current interview round, our questioning requests a response addressing how the broader “industry” perceives ROI, contrasting with the prior survey, where the inquiry was implicitly directed toward respondents’ companies and customers. Also, it is worth noting that the earlier interviews featured a specific count of individuals with dedicated sales-oriented roles. This difference in mentions may be attributed to the question’s framing.

## Respondent View—ROI of AI in 3 to 5 years

The five-year vision for AI adoption continues to be very optimistic from the vendor perspective, expecting broader AI adoption, a greater breadth of AI solutions, and expansion to new forms of AI, including large language models (LLM) and other quantitative tools (e.g., radiomics). Vendors who participated in the survey that referenced having a reimbursement code as a source of ROI often have AI solutions for triage and strongly believe that AI will be a standard of care within five years.

The ML Committee found this optimistic future vision in contrast to the limited respondents who were AI adopters. These adopters were less confident about the uptake of AI based on trust, transparency, and cost/ROI. Table 12 summarizes the areas where respondents repeatedly mentioned potential use cases where ROI could be attained. The survey was specific to radiology, so these do not address institutional opportunities for ROI.

**Table 12:** AI user responses on sources of ROI

**Mentions of ROI in 3–5 years**	**Count of response**
**Disease detection**	9
**Disease detection and triage**	6
**Productivity—reporting**	2
**Quality and triage**	1
**Triage**	7
**Not sure**	3

Elaborating on the content in Table 12, there was also mention of how some AI may impact the labor shortage for imaging services, aiding in modality throughput, and expressing a sentiment that with the assistance of AI, screening studies could be read by non-radiologists, although this was not a consensus.

Given the multitude of ways AI is being applied (e.g., in modalities, in PACS, and AI platforms), the observations from the interviews gave the sense that AI is already here through a transparent or default inclusion in products. With that, their 5-year outlook was more of the same.

When looking at the longer-term horizon for AI adoption, respondents focused on the barriers to trust in AI and the impact of the FDA filing. Skeptical and critical remarks about barriers to adoption were numerous, highlighting some repetitive themes, including the following:

- Several interviews mentioned CAD in mammography as a complex adoption experience for what is now AI. The breadth of adoption was believed to be driven by the substantial reimbursement and yet somewhat uneven experiences with clinical outcomes. Non-CAD vendors shared that a bias against AI adoption came from stories or direct experiences where there was a volume of CAD false positives that negatively impacted productivity. As a result, the 2023 survey still mentioned skepticism toward the general adoption of AI beyond very large enterprises.
- Descriptions of overhyping to the complexity of decisions regarding which AI to deploy on which platform (e.g., PACS, third party, or AI-specific platform). There was no mention of the April, 2023, release of Predetermined Change Control Plans (“PCCP”) from the FDA, which allows for revisions of AI without submission.^4^ However, as each AI algorithm is registered with the FDA for a single-diagnostic function rather than each AI tool having a broader application to a study makes the adoption more complex (as in a panel of AI from a single vendor versus a consensus of multiple AI for a single study). Specifically, the application of AI tools needs to process studies across many AI tool, perhaps on different inference platforms, for a single study—which can raise costs and potentially impact interpretation time.

## Respondents View—Barriers to AI Adoption

In 2021, respondents shared barriers they heard or saw, including getting AI to market and the impact of standards. These barriers/risks differed in 2023, with far less mention of standards.

In 2023, our respondents expect consolidation to occur. They identified an evolution in the market with large, more successful vendors emerging and separating themselves from the numerous small vendors. Given the difficulty of competing with well-funded large players and core modality companies, this market will likely consolidate as small, single-purpose AI cannot outcompete a more comprehensive set of solutions from the larger companies or AI platforms hosting multiple AI models.

The respondent mentioned the coming and going of several AI vendors as a risk to adoption and the concept of trust in AI. Several mentioned that fixed releases of AI tools and resubmission requirements need to consider the opportunity for dynamic training of AI models to better adjust to the local specifics of the patient population.^5^

The impact of AI adoption outside the USA was a frequently mentioned discriminator. National public health offers a high-volume but bureaucratic customer model for AI, given its greater scale and, thus, potential ROI. Respondent’s further voiced that given less complex regulatory approvals for CE certification, it was easier to register comprehensive AI tools and with the volume of the public health market represents a stronger opportunity to fuel AI adoption.^6^

Tables 13 and 14 show consistency across the surveys of the perceived barriers to adoption even with the expanded pool of respondents. At the time of the survey, there were several efforts to offer standards for integration, as shown by the workflow definitions by IHE.^7^ However, the respondents in the 2023 survey did not mention these, and they still perceived standards as an issue.

**Table 13:** 2021 responses to barriers to AI adoption by title of respondent

**Mention of barriers to adoption in 2021 by role**
**Role: Business development**
**Lack of a standard for AI to add to reports**
**Lack of standardized reports for data mining**
**No standard for integration**
**Not clear that workflow for AI is defined**
**Lack of standards for adoption and adapting AI**
**Role: Chief product officer/manager**
**Non-standard data curation**
**Deployment methodology**
**How to democratize data**
**Lack of standard for integration**
**Lack of standard data sets for evaluation**
**Role: President/sales**
**DICOM is not the format for AI**
**How to evaluate AI**
**Standards get AI output into reports**
**Standardizing reports for data mining**
**Integration efforts today are ahead of standards**
**Role: VP sales**
**AI is not necessary but there**
**No clear workflow defined**
**PACS is the integration point for AI**

**Table 14:** 2023: What are the barriers to adoption

**Mention of barriers to adoption?** **(multiple entries allowed)**	
**Need for more data and more diverse data**	12
**Lack of standard validation**	8
**Integration challenges**	8
**Lack of trust in AI**	6
**Lack of proof of performance**	4
**Lack of reimbursement**	4
**Lack of regulation**	3
**Lack of performance of AI**	3
**Lack of standards**	3
**Lack of ROI**	3
**Cost of AI**	2
**Lack of technical support**	1

In 2023, the barriers to adoption added a different perspective in the mention of transparency of how AI delivers its results and how those results are measured consistently. This accentuation on transparency was more of the perspective of what is important to the consumer of AI.

In the interviews, queries were asked about promoting the adoption of AI, the barriers, and what could remove the barriers. AI vendors were vocal about needing more data to build out the art of the possible, but as shown in Table 14, some concerns are more about what we have been trustworthy and transparent about how the AI is making decisions. These concerns remain consistent across the two surveys tracked in 2021 and 2023.

Table 15 describes a follow-up ask during the 2023 interviews, which provides guidance on how the respondents see the market conditions changing and how they are developing tools along with adopters to address the general lack of things above. Respondents mentioned that the barrier of not having data is possibly being addressed as more opportunities to leverage clinical data partnerships are becoming available.

**Table 15:** 2023: how can these barriers be reduced?

**How can these barriers be reduced?** ***(multiple choice)***	
**AI vendors access to more data and more diverse data**	15
**AI performance tracking mechanisms**	9
**Reimbursement**	7
**Outcome studies/papers**	6
**Regulatory compliance requirements**	2

## Conclusion

The adoption of artificial intelligence remains a pivotal issue in diagnostic imaging. Our survey continues to show enthusiasm for collaborating on accelerating the adoption across providers, while life science is mentioned by some as an area of interest. The SIIM committee anticipates that repeated surveys of the market vendors and AI adopters will continue to describe progress in removing the barriers to wider access to these important tools for productivity and, ultimately, patient care.

## Data Availability

No specific data is available to be shared and surveys were conducted under confidentiality.

## Footnotes

1 The SIIM Machine Learning (ML) Industry Liaison Committee, a committee of the SIIM Machine Learning Committee, comprises radiologists, technologists, industry analysts, and vendors.

2 Current Procedural Terminology (CPT^®^) by American Medical Association.

3 CMS stands for Centers for Medicare and Medicaid Services, the federal agency that runs the Medicare, Medicaid, and Children’s Health Insurance Programs, and the federally facilitated Marketplace.

4 Food and Drug Administration (.gov), Predetermined Change Control Plans for Machine Learning-Enabled Medical Devices: Guiding Principles.

5 The committee recognizes that the FDA released a new policy on iterative releases after this survey was conducted.

6 This respondent opinion is echoed in https://operonstrategist.com/ce-mark-vs-fda-approval-for-medical-devices, “…, getting CE Mark approval is typically regarded as being simpler than getting FDA approval.. as the FDA 510(k) procedure requires proving a device’s “substantial equivalence” to an already-available device to show that it is safe and effective…”.

7 Integrating the Healthcare Enterprise (IHE).

